# Topological superconductors from one-dimensional periodically modulated Majorana chains

**DOI:** 10.1038/s41598-017-09160-x

**Published:** 2017-08-23

**Authors:** Yang Lin, Weichang Hao, Mei Wang, Jianqiang Qian, Huaiming Guo

**Affiliations:** 1 Department of Physics, Key Laboratory of Micro-Nano Measurement-Manipulation and Physics (Ministry of Education), Beihang University, Beijing, 100191 China; 2 Beijing Computational Science Research Center, Beijing, 100089 China

## Abstract

By analogy to the topological models of fermions in one-dimensional periodically modulated lattices, we provide a systematic method to construct topological superconductors in BDI class. We then create superlattices of Majorana fermions to interpolate several Majorana chains, and realize topological superconductors with arbitrary winding numbers. Two kinds of chiral symmetries are identified in the systems with multiple chains. Of the two winding numbers associated to the chiral symmetries, one counts the number of zero-energy modes, while the other counts the difference of the numbers of *α*- and *β*-type Majorana zero states. We also show that one *α*- and one *β*-type Majorana zero modes collapse into fractional charged zero states when they are spatially intertwined. In the systems with odd number of chains, it induces topological superconductors with coexistence of fractional charged zero states and Majorana zero states. Finally by introducing symmetry breaking term, we present an intuitive explanation of the Z_2_ nature of the topological invariant in the D class.

## Introduction

Majorana fermions, which are their own antiparticles, have been a very active pursuit in condensed matter physics due to their fundamental significance and potential applications in topological quantum computation^1–4^. As pointed out by Kitaev, Majorana fermions can emerge on the boundaries of a one-dimensional (1D) spinless p-wave superconductor^5^. Recently the toy model has been engineered in different experimental setups and possible signatures of Majorana fermions have been observed^6–9^.

The search for Majorana fermions simulates theoretical constructions of topological superconductors from various approaches. The topological classification shows that there should be four classes of topological superconductors due to the different symmetries, i.e., BDI, CII, D and DIII^10^. The 1D Kitaev chain with complex (real) pairing belongs to the D (BDI) class. The ones belonging to other classes are attracting growing studies^11–14^. Besides the strict 1D system, it is suggested that 1D topological superconductors can be realized in narrow strips of 2D ones^15–18^. It is also found that topological superconductors can be constructed by simply adding superconducting pairing in 1D topological insulators, such as the Creutz model, the Su-Schrieffer-Hegger (SSH) model^19–22^. Another route to obtain unpaired Majorana fermions is from the cores of vortices in the two-dimensional *p*_*x*_ + *ip*_*y*_ topological superconductors^23, 24^. Although there exist experimental hurdles for such kind of proposals, their advantages are obvious, such as the realization of the non-abelian statistics. Specially in the setups, we can extend from single Majorana zero mode to Majorana lattices, which will provide the opportunity to study systems with Majorana fermions as the basic particles^25, 26^.

Motivated by the progress, we figure out a natural way to construct topological superconductors, i.e., simply replacing fermion operators with Majorana operators in the known topological models of fermions. If the resulting systems with Majorana operators hold on, the boundary states exist and are in terms of Majorana operators. Then the systems should be topological superconductors. The approach seems reasonable, but it has a problem since not all fermionic terms are realistic under such simple replacements. For example, there are no on-site terms with Majorana operators in that *γ* ^†^ *γ* = 1 (*γ* is Majorana operator). Also for the hopping terms, the amplitudes should only be imaginary. Though the approach doesn’t work for general cases, it can be applied to specific topological systems with only pure real or imaginary hopping terms. Fortunately we indeed have such kind of topological systems, one of which is 1D lattices with periodically modulated nearest-neighbor (NN) hoppings (also known as off-diagonal Harper model)^27, 28^.

In the paper, we start from the topological models of fermions in one-dimensional periodically modulated lattices. By simply replacing fermion operators with Majorana operators, we obtain a class of 1D topological supercondcutors in BDI class. While the above methods generate topological superconductors with the topological invariant 1, the topological systems with arbitrary topological invariants can be obtained by interpolating multiple Majorana chains. Such systems exhibit rich physical properties. Two kinds of chiral symmetries are identified, corresponding to which two winding numbers are defined. It is found that one winding number counts the number of zero-energy modes, while the other counts the difference of the numbers of *α*- and *β*-type Majorana zero states. The physical meaning behind the latter winding number is the destruction of different type Majorana zero states when they are spatially interwined. The mechanism induces the realization of topological superconductors with coexistence of fractional charged zero states and Majorana zero states. By changing the topological class of the system from BDI to D, an intuitive explanation of the reduction of the topological invariant is presented.

## Results and Discussions

### Majorana fermions in 1D periodically modulated chains

Consider Majorana fermions in 1D chains with periodically modulated hopping amplitudes, which is described by the following Hamiltonian,

1 $$H=i\sum _{j=1}^{L}\,({t}_{2j-1}{\alpha }_{j}{\beta }_{j}+{t}_{2j}{\beta }_{j}{\alpha }_{j+1}),$$

where ${\alpha }_{j}={c}_{j}+{c}_{j}^{\dagger }$, ${\beta }_{j}=\frac{{c}_{j}-{c}_{j}^{\dagger }}{i}$ are Majorana operators and ${c}_{j}({c}_{j}^{\dagger })$ is the fermion annihilation (creation) operator; the hopping amplitudes are periodically modulated, i.e., *t*_*j*_ = *t*_*j*+*T*_ with *T* the period. Since the system has Majorana fermions in sequence, *T* should be even. By analogy to 1D periodically modulated lattices of fermions, it is expected that the above models support topological superconductors with unpaired Majorana fermions.

Firstly we study the case of *T* = 2. Passing to the fermionic basis, we have

2 $${H}_{1}=\sum _{j}\,[(-{t}_{2}{c}_{j}^{\dagger }{c}_{j+1}+{t}_{2}{c}_{j}{c}_{j+1}+{\rm{H}}\mathrm{.}{\rm{c}}\mathrm{.)}+2{t}_{1}({n}_{j}-\frac{1}{2})]\mathrm{.}$$

In the momentum space and under the basis ${\psi }_{k}={\{{c}_{\mathrm{1,}k},{c}_{\mathrm{1,}-k}^{\dagger }\}}^{T}$, it is written as ${H}_{1}={\sum }_{k}\,{\psi }_{k}^{\dagger }{ {\mathcal H} }_{1}(k){\psi }_{k}$, where ${ {\mathcal H} }_{1}(k)=(-{t}_{2}\,\cos \,k+{t}_{1})\,{\sigma }_{z}+{t}_{2}\,\sin \,k{\sigma }_{y}$ with *σ*_*j*_(*j* = *x*, *y*, *z*) the Pauli matrices. The energy spectrum is directly obtained: $E(k)=\pm \sqrt{{(-{t}_{2}\cos k+{t}_{1})}^{2}+{({t}_{2}\sin k)}^{2}}$. The gap closes when |*t*_1_| = |*t*_2_|. Since the parameters of the above Hamiltonian are all real, it has time-reversal symmetry ${\mathscr{T}}\,{ {\mathcal H} }_{1}(k){{\mathscr{T}}}^{-1}={ {\mathcal H} }_{1}(-k)$ with the time-reversal operator ${\mathscr{T}}={\mathscr{K}}$ (the complex conjugate). The Hamiltonian also has chiral symmetry $C\,{ {\mathcal H} }_{1}(k){C}^{-1}=-{ {\mathcal H} }_{1}(k)$ with the chiral operator *C* = *σ*_*x*_. So the system belongs to the BDI class, whose topological invariant is an integer. We can transform the chiral operator to the diagonal form with an unitary transformation *U*, i.e., *U* ^−1^ *CU* = *σ*_*z*_. Meanwhile since the Hamiltonian anticommunicates with the chiral symmetry, it becomes off-diagonal under the same transformation,

$$U\, {\mathcal H} \,(k){U}^{-1}=(\begin{array}{cc}0 & V(k)\\ V{(k)}^{\dagger } & 0\end{array})\mathrm{.}$$

The topological invariant is the winding number of Det(*V*), which is defined as refs 29–31,

3 $$N=-{\int }_{-\pi }^{\pi }\,\frac{dk}{2\pi i}{\partial }_{k}\,{\rm{lnDet}}(V\mathrm{).}$$

**Figure 1:**
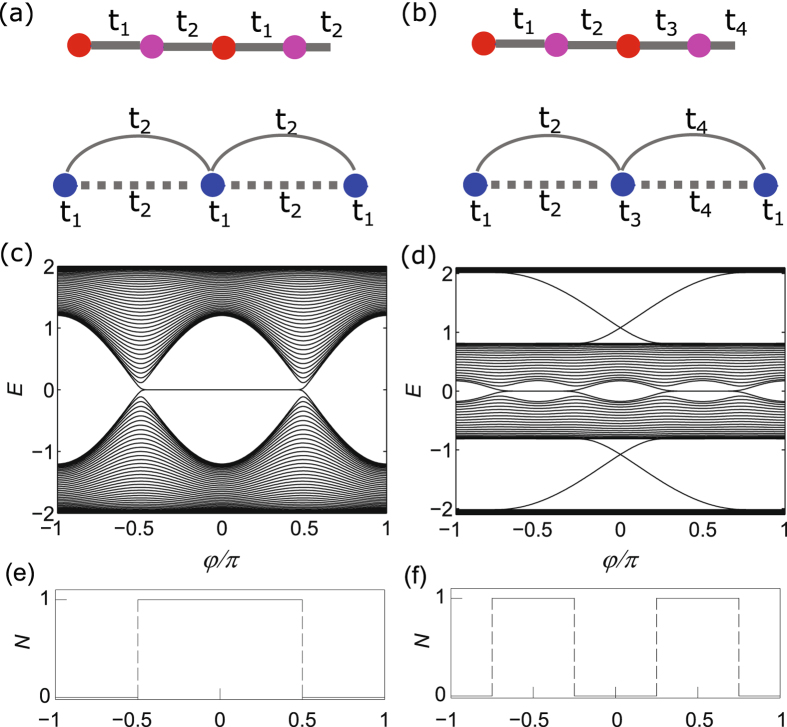
Schematic illustration of the model Eq. (1) (upper) with the period: (**a**) *T* = 2; (**b**) *T* = 4. The red (pink) circles represent Majorana fermions *α* (*β*). The lower ones schematically illustrate the corresponding models in terms of fermions (blue circles). The energy spectrum on an open chain as a function of *φ*: (**c**) *T* = 2; (**d**) *T* = 4. The winding number as a function of *φ*: (**e**) *T* = 2; (**f**) *T* = 4. The parameter *λ* = 0.6 is used.

For the above Hamiltonian ${\mathcal H} (k)$, the transformation matrix is,

4 $$U=\frac{1}{\sqrt{2}}(\begin{array}{cc}1 & 1\\ -i & i\end{array}),$$

and the resulting off-diagonal element is *V*(*k*) = *i*(*t*_1_ − *t*_2_ *e* ^−*ik*^). Its winding number is *N* = 1 for |*t*_1_| < |*t*_2_|, while *N* = 0 for |*t*_1_| > |*t*_2_|. We can interchange the Majorana operators *α*, *β* in Fig. 1(a). The resulting Hamiltonian in the fermionic basis still has the form of Eq. (2), except the signs of the chemical potentials and the NN hopping amplitudes. Following the above procedure, we have *V*′(*k*) = −*i*(*t*_1_ − *t*_2_ *e* ^*ik*^). The winding number is *N* = −1 for |*t*_1_| < |*t*_2_|. The sign of the winding number is from the winding direction of Det(*V*) and reflects the type of the unpaired Majorana fermion at the boundary.

Due to bulk-boundary correspondence, there appear zero boundary modes on open chains in the topological phases. To give concrete examples, we take the cosine modulations ${t}_{j}=t[1+\lambda \,\cos \,(\frac{2\pi j}{T}+\phi )]$ with phase factor *φ* and *t* = 1 taken as the energy unit. We calculate the energy spectrum under open boundary condition as a function of *φ*, which is shown in Fig. 1. The topological phases appears in alternating regions separated by the gapless points ${\phi }_{n}=\frac{2\pi }{T}(n+\frac{1}{2})$, $n=0,\ldots ,T-1$ and have the winding number *N* = 1. In the topological phases, there appears a pair of zero boundary modes, which are Majorana fermions. We also study the case with *T* = 4, which are the SSH model with *p*-wave superconduncting pairings, and the results are similar. More generally, the above procedure is applicable to any even *T* and the resulting models remain in extended parameters’ regions.

Thus we provide a systematic method to construct topological superconductors in BDI class with |*N*| = 1 and a pair of Majorana zero modes. It is desirable to study cases with arbitrary winding numbers. A direct way is to simply put the above 1D Hamiltonians together. Instead to make the system coupled, we create superlattices of Majorana fermions to interpolate several Majorana chains.

### Destruction of two Majorana zero states with opposite chiralities

**Figure 2:**
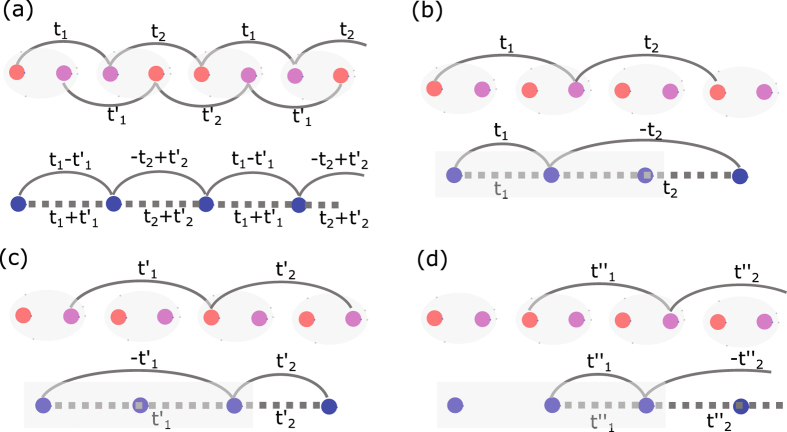
Schematic illustration of the models. (**a**) Eq. (5) (upper) and Eq. (6) (lower). The system of three Majorana chains described by Eqs (12) and (13) is shown separately in (**b**–**d**). The red (pink) circles represent Majorana fermions *α* (*β*) and the blue circles represent fermions.

We firstly consider the case of two Majorana chains with the configuration shown in Fig. 2(a), which is described by the Hamiltonian,

5 $$\begin{array}{rcl}{H}_{2}^{\mathrm{(2)}} & = & i\sum _{j=1}^{L}\,[({t}_{1}{\alpha }_{2j-1}{\beta }_{2j}+{t}_{2}{\beta }_{2j}{\alpha }_{2j+1})\\  &  & +\,({t}_{1}^{^{\prime} }{\beta }_{2j-1}{\alpha }_{2j}+{t}_{2}^{^{\prime} }{\alpha }_{2j}{\beta }_{2j+1})].\end{array}$$

Transforming it to the fermionic basis we have,

6 $$\begin{array}{rcl}{H}_{2}^{\mathrm{(2)}} & = & \sum _{j=1}^{L}\,[({t}_{1}-{t}_{1}^{^{\prime} })\,{c}_{2j-1}^{\dagger }{c}_{2j}-({t}_{2}-{t}_{2}^{^{\prime} })\,{c}_{2j}^{\dagger }{c}_{2j+1}\\  &  & +\,({t}_{1}+{t}_{1}^{^{\prime} })\,{c}_{2j-1}{c}_{2j}+({t}_{2}+{t}_{2}^{^{\prime} })\,{c}_{2j}{c}_{2j+1}+{\rm{H}}.{\rm{c}}\mathrm{.}].\end{array}$$

Though the system is decoupled with Majorana operators, it is not in the fermionic basis. In the momentum space and under the basis ${\psi }_{k}={\{{c}_{\mathrm{1,}k},{c}_{\mathrm{1,}-k}^{\dagger },{c}_{\mathrm{2,}k},{c}_{\mathrm{2,}-k}^{\dagger }\}}^{T}$, it is written as ${H}_{2}^{\mathrm{(2)}}=\frac{1}{2}{\sum }_{k}\,{\psi }_{k}^{\dagger }{ {\mathcal H} }_{2}^{\mathrm{(2)}}\,(k)\,{\psi }_{k}$ with

7 $${ {\mathcal H} }_{2}^{\mathrm{(2)}}\,(k)=(\begin{array}{cccc}0 & 0 & w & z\\ 0 & 0 & -z & -w\\ {w}^{\ast } & -{z}^{\ast } & 0 & 0\\ {z}^{\ast } & -{w}^{\ast } & 0 & 0\end{array}),$$

where $w={t}_{1}-{t}_{1}^{^{\prime} }-({t}_{2}-{t}_{2}^{^{\prime} })\,{e}^{-ik}$, $z=-({t}_{1}+{t}_{1}^{^{\prime} })+({t}_{2}+{t}_{2}^{^{\prime} })\,{e}^{-ik}$. Its energy spectrum is, *E*(*k*) = ±|*w* ± *z*|. The gap closes when |*t*_1_| = |*t*_2_| or $|{t}_{1}^{^{\prime} }|=|{t}_{2}^{^{\prime} }|$. ${ {\mathcal H} }_{2}^{\mathrm{(2)}}\,(k)$ can be writen compactly in terms of Dirac matrices,

8 $$\begin{array}{rcl}{ {\mathcal H} }_{2}^{\mathrm{(2)}}(k) & = & [{t}_{1}-{t}_{1}^{^{\prime} }-({t}_{2}-{t}_{2}^{^{\prime} })\,\cos \,k]\,{\tau }_{x}\otimes {\sigma }_{z}\\  &  & +\,[{t}_{1}+{t}_{1}^{^{\prime} }-({t}_{2}+{t}_{2}^{^{\prime} })\,\cos \,k]\,{\tau }_{y}\otimes {\sigma }_{y}\\  &  & -\,({t}_{2}-{t}_{2}^{^{\prime} })\,\sin \,k{\tau }_{y}\otimes {\sigma }_{z}+({t}_{2}+{t}_{2}^{^{\prime} })\,\sin \,k{\tau }_{x}\otimes {\sigma }_{y},\end{array}$$

The above real Hamiltonian has chiral symmetry ${C}_{2}{ {\mathcal H} }_{2}^{\mathrm{(2)}}\,(k){C}_{2}^{-1}=-{ {\mathcal H} }_{2}^{\mathrm{(2)}}\,(k)$ with the chiral operator *C*_2_ = *I* ⊗ *σ*_*x*_. So it belongs to the BDI class. By a unitary transformation

9 $${U}_{2}=\frac{1}{\sqrt{2}}\,(\begin{array}{cccc}1 & 1 & 0 & 0\\ 0 & 0 & 1 & 1\\ -i & i & 0 & 0\\ 0 & 0 & -i & i\end{array}),$$

we have ${U}_{2}{C}_{2}{U}_{2}^{-1}={\tau }_{z}\otimes I$, and ${U}_{2}{ {\mathcal H} }_{2}^{\mathrm{(2)}}\,(k)\,{U}_{2}^{-1}$ is in a block off-diagonal form with the upper block,

10 $${V}_{2}(k)=2i(\begin{array}{cc}0 & {t}_{1}-{t}_{2}{e}^{-ik}\\ -{t}_{1}^{^{\prime} }+{t}_{2}^{^{\prime} }{e}^{ik} & 0\end{array})\mathrm{.}$$

Its determinant is ${\rm{Det}}(V)=-\mathrm{4(}{t}_{1}-{t}_{2}{e}^{-ik})\,({t}_{1}^{^{\prime} }-{t}_{2}^{^{\prime} }{e}^{ik})$, thus the winding number is *N* = *N*_1_ + *N*_2_ with *N*_1_, *N*_2_ the winding number of individual Majorana chain. Depending on the parameters, the winding number can be *N* = 0, ±1 (see Table 1).

**Table 1:** The winding numbers associated with the two chiral symmetries: (a) the case of two Majorana chains; (b) the case of three Majorana chains with $|{t}_{1}^{^{\prime\prime} }| < |{t}_{2}^{^{\prime\prime} }|$ [the case with $|{t}_{1}^{^{\prime\prime} }| > |{t}_{2}^{^{\prime\prime} }|$ is the same with (a)].

**(a)**		
	|*t*_1_| < |*t*_2_|	|*t*_1_| > |*t*_2_|
$|{t}_{1}^{^{\prime} }| < |{t}_{2}^{^{\prime} }|$	*N* = 0, *N*′ = 2	*N* = −1, *N*′ = 1
$|{t}_{1}^{^{\prime} }| > |{t}_{2}^{^{\prime} }|$	*N* = 1, *N*′ = 1	*N* = 0, *N*′ = 0
**(b)**		
	|*t*_1_| < |*t*_2_|	|*t*_1_| > |*t*_2_|
$|{t}_{1}^{^{\prime} }| < |{t}_{2}^{^{\prime} }|$	*N* = 1, *N*′ = 3	*N* = 0, *N*′ = 2
$|{t}_{1}^{^{\prime} }| > |{t}_{2}^{^{\prime} }|$	*N* = 2, *N*′ = 2	*N* = 1, *N*′ = 1

It is noticed that the Hamiltonian has been block off-diagonal in its original form, which means that by putting two chains together the combined system obtains an additional chiral symmetry with the chiral operator ${C}_{2}^{^{\prime} }={\tau }_{z}\otimes I$. The topological invariant is the winding number of ${\rm{Det}}(V^{\prime} )=\mathrm{4(}{t}_{1}-{t}_{2}{e}^{-ik})\,({t}_{1}^{^{\prime} }-{t}_{2}^{^{\prime} }{e}^{-ik})$ with *V*′(*k*) the upper off-diagonal block of ${ {\mathcal H} }_{2}^{\mathrm{(2)}}$. Thus we identify a system in BDI class, but with two kinds of chiral symmetries and characterized by two winding numbers. It is interesting to ask what are the meanings of the winding numbers.

**Figure 3:**
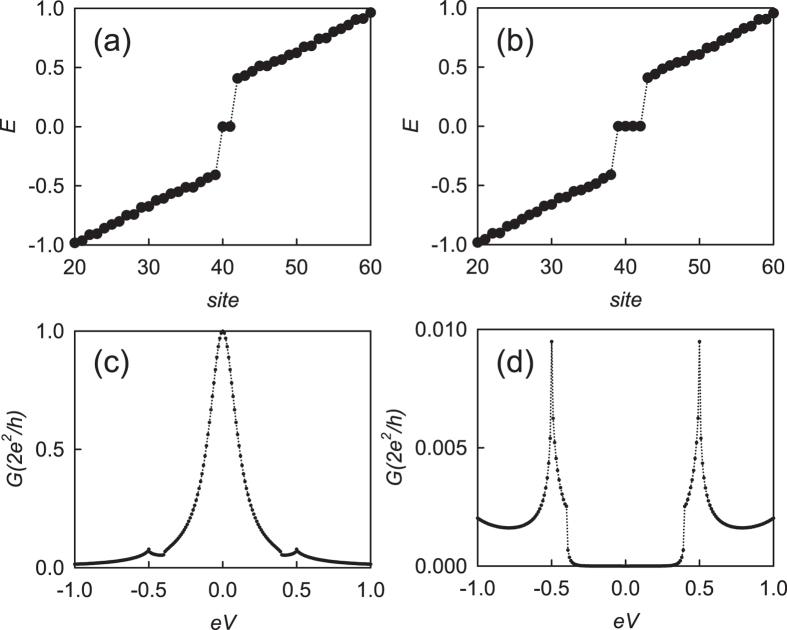
The open energy spectrum for the case of two Majorana chains: (**a**) ${t}_{1}^{^{\prime} }=-0.8,\,{t}_{2}^{^{\prime} }=-0.4$ with the winding number *N* = 1, *N*′ = 1; (**b**) ${t}_{1}^{^{\prime} }=-0.4,\,{t}_{2}^{^{\prime} }=-0.8$ with the winding number *N* = 0, *N*′ = 2. (**c**,**d**) the conductance of a normal lead/topological superconductor junction as a function of voltage with the topological superconductors described in (**a**,**b**), respectively. In (**c**), the width of the peak is controlled by the transparency of the junction, i.e., the hopping *t*_*c*_ between the normal and superconducting sides of the junction. The width increases as *t*_*c*_ is decreased, which is similar to the case of unconventional superconductor^33^. The other parameters are *t*_1_ = 0.5, *t*_2_ = 1.

It is direct to see that the winding number *N*′ associated with the chiral symmetry ${C}_{2}^{^{\prime} }$ equals the number of zero-energy states, as shown in Fig. 3(a,b). We also notice the winding number *N* associated with the chiral symmetry *C*_2_ equals the difference of the numbers of *α*- and *β*-type Majorana zero states. It is noted that the topological invariant *N* has already been given in the form of index theorem^32^, which has exactly the same physical meaning with ours. The physical meaning of the winding number *N* can be interpreted as the destruction of Majorana zero states with opposite chiralities. The effect is particularly evident when the Majorana zero states are spatially intertwined. In the following we study the effect from the case with *N* = 0, *N*′ = 2, which has one *α*- and *β*-type Majorana zero states at each boundary.

**Figure 4:**
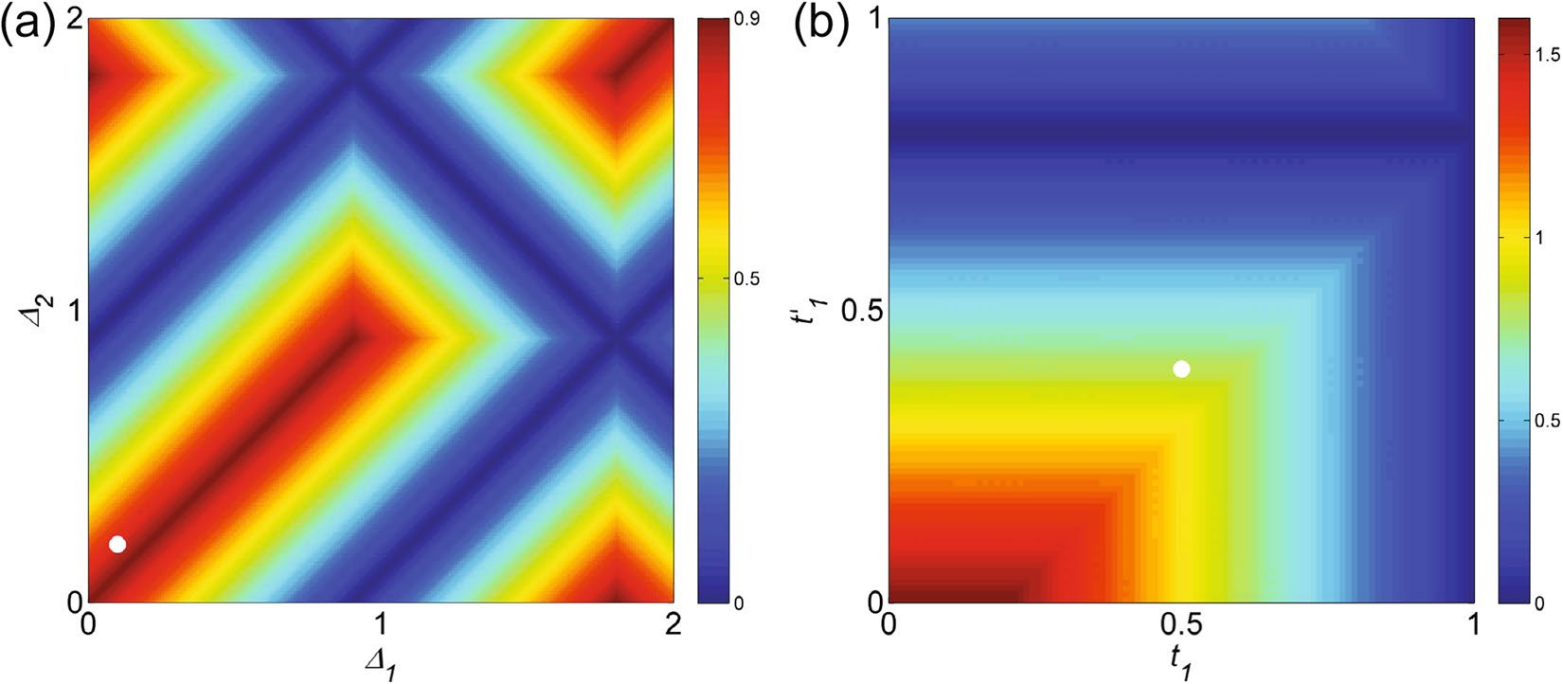
The gap for the case of two Majorana chains: (**a**) in the (Δ_1_, Δ_2_) plane; (**b**) in the $({t}_{1},\,{t}_{1}^{^{\prime} })$ plane. The parameters are *t*_1_ = 0.5, *t*_2_ = 1, ${t}_{1}^{^{\prime} }=-0.4,\,{t}_{2}^{^{\prime} }=-0.8$, when the topological phase is with *N* = 0, *N*′ = 2 and is denoted by the white dots in the figures.

Firstly we show the abvoe phase is adiabatically connected to non-superconducting case. We change the pairing amplitudes in Eq. (6) $({t}_{1}+{t}_{1}^{^{\prime} })$, $({t}_{2}+{t}_{2}^{^{\prime} })$ to Δ_1_, Δ_2_ and map the gap of the system in the (Δ_1_, Δ_2_) plane. As shown in Fig. 4(a), the white dot corresponding to the topological phase with *N* = 0, *N*′ = 2 is adiabatically connected to non-superconducting case, which is the SSH model. Then we study the property of the zero-energy quasiparticles. We can consider the limit case *t*_1_ = 0, ${t}_{1}^{^{\prime} }=0$, which is adiabatically connected to the general cases [see Fig. 4(b)]. The limit case can be exactly solved since the end sites become isolated and the system can be decoupled to central part and the end sites. The states on one end site can be |0〉, |1〉 (0, 1 the number of electron) with zero energy. Suppose the ground-state wavefunction is |*GS*〉 and the ground-state wavefunction of the whole system can be obtained by a direct product: ${|GS\rangle }_{T}={|\mathrm{0(1)}\rangle }_{L}\otimes |GS\rangle \otimes {|\mathrm{0(1)}\rangle }_{R}$ [*L*(*R*) denote the left (right) end sites], which is four-fold degenerate. Thus we have four zero-energy quasiparticle operators ${C}_{1},\,{C}_{1}^{\dagger },\,{C}_{N},\,{C}_{N}^{\dagger }$, which are just electrons or holes. The zero-energy modes are just like those in the SSH model and we can term them as fractional charged zero states. Thus we show that the two Majorana zero states *α*, *β* at each end collapse into an electron or hole.

The destruction of two Majorana zero states with opposite chiralities results in the suppression of local Andreev reflection in a normal lead/topological superconductor junction. We calculate the differential conductance as a function of the bias voltage^34, 35^. The zero bias conductance is calculated as ref. 35,

11 $$\begin{array}{rcl}\,\,\,G & = & \frac{{e}^{2}}{h}Tr[I-sgn(\alpha ){S}_{ij}^{e\alpha }{(E)}^{\dagger }\,{S}_{ij}^{e\alpha }(E)],\\ {S}_{ij}^{\alpha \beta } & = & -{\delta }_{ij}{\delta }_{\alpha \beta }+i{[{{\rm{\Gamma }}}_{i}^{\alpha }]}^{\mathrm{1/2}}\,{G}^{r}{[{{\rm{\Gamma }}}_{i}^{\beta }]}^{\mathrm{1/2}}\mathrm{.}\end{array}$$

${S}_{ij}^{\alpha \beta }$ is an element of the scattering matrix which denotes the scattering amplitude of a *β* particle from lead *j* to a *α* particle in lead *i*, where *i*, *j* = 1, *or*, 2, and *α*(*β*) denotes electron (hole). As shown in Fig. 3(c), when there is one Majorana zero state at the end, the resonant local Andreev reflection happens and there appears zero bias conductance peak with the height $G=\frac{2{e}^{2}}{h}$. However the zero bias conductance peak vanishes in the presence of two spatially interwined Majorana zero states with opposite chiralities [see Fig. 3(d)], which is consistent with the fact that the two Majorana zero states are interwined^36^.

### Coexistence of fractional charged and Majorana zero states

Next we consider the case of three Majorana chains with the configuration shown in Fig. 2(b–d). It is described by the Hamiltonian,

12 $$\begin{array}{rcl}{H}_{3}^{\mathrm{(2)}} & = & i\sum _{j=1}^{L}\,[({t}_{1}{\alpha }_{3j-2}{\beta }_{3j-1}+{t}_{2}{\beta }_{3j-2}{\alpha }_{3j+1})\\  &  & +\,({t}_{1}^{^{\prime} }{\beta }_{3j-2}{\alpha }_{3j}+{t}_{2}^{^{\prime} }{\alpha }_{3j}{\beta }_{3j+1})\\  &  & +\,({t}_{1}^{^{\prime\prime} }{\alpha }_{3j-1}{\beta }_{3j}+{t}_{2}^{^{\prime\prime} }{\beta }_{3j}{\alpha }_{3j+2})]\mathrm{.}\end{array}$$

In the fermionic basis it becomes,

13 $$\begin{array}{rcl}{H}_{3}^{\mathrm{(2)}} & = & \sum _{j=1}^{L}\,[{t}_{1}{c}_{3j-2}^{\dagger }{c}_{3j-1}+{t}_{1}^{^{\prime\prime} }{c}_{3j-1}^{\dagger }{c}_{3j}+{t}_{2}^{^{\prime} }{c}_{3j}^{\dagger }{c}_{3j+1}\\  &  & +\,{t}_{1}{c}_{3j-2}{c}_{3j-1}+{t}_{1}^{^{\prime\prime} }{c}_{3j-1}{c}_{3j}+{t}_{2}^{^{\prime} }{c}_{3j}{c}_{3j+1}\\  &  & -\,{t}_{1}^{^{\prime} }{c}_{3j-2}^{\dagger }{c}_{3j}-{t}_{2}{c}_{3j-1}^{\dagger }{c}_{3j+1}-{t}_{2}^{^{\prime\prime} }{c}_{3j}^{\dagger }{c}_{3j+2}\\  &  & +\,{t}_{1}^{^{\prime} }{c}_{3j-2}{c}_{3j}+{t}_{2}{c}_{3j-1}{c}_{3j+1}+{t}_{2}^{^{\prime\prime} }{c}_{3j}{c}_{3j+2}+{\rm{H}}\mathrm{.}{\rm{c}}\mathrm{.}]\end{array}$$

In the momentum space and under the basis ${\psi }_{k}={\{{c}_{\mathrm{1,}k},{c}_{\mathrm{1,}-k}^{\dagger },{c}_{\mathrm{2,}k},{c}_{\mathrm{2,}-k}^{\dagger },{c}_{\mathrm{3,}k},{c}_{\mathrm{3,}-k}^{\dagger }\}}^{T}$, we have,

14 $${ {\mathcal H} }_{3}^{\mathrm{(2)}}=(\begin{array}{ccc}0 & {h}_{+} & {h}_{-}^{^{\prime} }\\ {h}_{+}^{\dagger } & 0 & {h}_{+}^{^{\prime\prime} }\\ {h}_{-}^{^{\prime} \dagger } & {h}_{+}^{^{\prime\prime} \dagger } & 0\end{array})$$

where,

15 $${h}_{\pm }^{\#}=(\begin{array}{cc}\pm ({t}_{1}^{\#}-{t}_{2}^{\#}{e}^{-ik}) & -({t}_{1}^{\#}-{t}_{2}^{\#}{e}^{-ik})\\ ({t}_{1}^{\#}-{t}_{2}^{\#}{e}^{-ik}) & \mp ({t}_{1}^{\#}-{t}_{2}^{\#}{e}^{-ik})\end{array})$$

with # representing the symbols ‘′’, ‘″’ or none.

The above Hamiltonian is real and has the chiral symmetry ${C}_{3}{ {\mathcal H} }_{3}^{\mathrm{(2)}}\,(k){C}_{3}^{-1}=-{ {\mathcal H} }_{3}^{\mathrm{(2)}}\,(k)$ with the chiral operator *C*_3_ = diag{*σ*_*x*_, *σ*_*x*_, *σ*_*x*_}. It belongs to the BDI class. The unitary transformation associated with the chiral symmetry *C*_3_ is

$${U}_{3}=\frac{1}{\sqrt{2}}(\begin{array}{cccccc}1 & 1 & 0 & 0 & 0 & 0\\ 0 & 0 & 0 & 0 & 1 & 1\\ 0 & 0 & 1 & 1 & 0 & 0\\ 0 & 0 & -i & i & 0 & 0\\ -i & i & 0 & 0 & 0 & 0\\ 0 & 0 & 0 & 0 & -i & i\end{array}),$$

under which ${ {\mathcal H} }_{3}^{\mathrm{(2)}}$ is block off-diagonal with the upper block,

$${V}_{3}(k)=2i(\begin{array}{ccc}{t}_{1}-{t}_{2}{e}^{-ik} & 0 & 0\\ 0 & -({t}_{1}^{^{\prime} }-{t}_{2}^{^{\prime} }{e}^{ik}) & 0\\ 0 & 0 & {t}_{1}^{^{\prime\prime} }-{t}_{2}^{^{\prime\prime} }{e}^{-ik}\end{array})\mathrm{.}$$

Its determinant is ${\rm{Det}}({V}_{3})=8i({t}_{1}-{t}_{2}{e}^{-ik})\,({t}_{1}^{^{\prime} }-{t}_{2}^{^{\prime} }{e}^{ik})\,({t}_{1}^{^{\prime\prime} }-{t}_{2}^{^{\prime\prime} }{e}^{-ik})$, so the winding number is the sum of those of individual chains, which counts the difference of the numbers of *α* and *β*-type Majorana fermions at the left end. Besides the above chiral symmetry, we also identify another chiral symmetry with the chiral operator ${C}_{3}^{^{\prime} }={\rm{diag}}\{I,{\sigma }_{x},-I\}$. With a transformation,

$${U}_{3}^{^{\prime} }=\frac{1}{\sqrt{3}}(\begin{array}{cccccc}\sqrt{3} & 0 & 0 & 0 & 0 & 0\\ 0 & \sqrt{3} & 0 & 0 & 0 & 0\\ 0 & 0 & 1 & 1 & 0 & 0\\ 0 & 0 & 0 & 0 & i & -i\\ 0 & 0 & -1 & 1 & -1 & -1\\ 0 & 0 & 1 & -1 & -1 & -1\end{array}),$$

we have ${U}_{3}^{^{\prime} }{C}_{3}^{^{\prime} }{U}_{3}^{^{\prime} -1}={\rm{diag}}\{1,1,1,-1,-1,-1\}$. And ${U}_{3}^{^{\prime} }{ {\mathcal H} }_{3}^{\mathrm{(2)}}{U}_{3}^{^{\prime} -1}$ is block off-diagonal with the upper block,

$${V}_{3}^{^{\prime} }(k)=(\begin{array}{ccc}0 & {v}_{1}(k) & -{v}_{2}(k)\\ 0 & {v}_{2}(k) & -{v}_{1}(k)\\ -2i({t}_{1}^{^{\prime\prime} }-{t}_{2}^{^{\prime\prime} }{e}^{-ik}) & 0 & 0\end{array})\mathrm{.}$$

where,

16 $$\begin{array}{l}{v}_{1}(k)=\frac{\sqrt{3}}{2}({t}_{2}{e}^{-ik}-{t}_{2}^{^{\prime} }{e}^{-ik}-{t}_{1}+{t}_{1}^{^{\prime} })\\ {v}_{2}(k)=\frac{\sqrt{3}}{2}({t}_{2}{e}^{-ik}+{t}_{2}^{^{\prime} }{e}^{-ik}-{t}_{1}-{t}_{1}^{^{\prime} }\mathrm{).}\end{array}$$

Its determinant is ${\rm{Det}}({V}_{3}^{^{\prime} })=-6i({t}_{1}-{t}_{2}{e}^{-ik})\,({t}_{1}^{^{\prime} }-{t}_{2}^{^{\prime} }{e}^{-ik})\,({t}_{1}^{^{\prime\prime} }-{t}_{2}^{^{\prime\prime} }{e}^{-ik})$. Its winding number counts the number of zero modes.

Thus the case of three Majorana chains is similar to the one of two Majorana chains. By tuning relative strengthes of the hopping amplitudes, we can obtain topological superconductors with different winding numbers, which are listed in Table 1. Of the various topological phases, we want to point out the case with *N* = 1, *N*′ = 3, in which each boundary has two *α* and one *β* Majorana zero states. As discussed in the previous section, the *α* and *β* Majorana zero states on the same site will intertwine and becomes fractional charged zero modes. Thus a topological phase with coexistence of fractional charged and Majorana zero states is realized.

### The reduction of the topological invariant from *Z* to *Z*_2_

We consider the terms breaking the symmetries of the system. Our discussions are based on the system with three Majorana chains since it exhibits more topological phases. We firstly add the NN hopping terms of Majorana fermions to the Hamiltonian Eq. (12), which is described by,

$${H}_{NN}=i\sum _{i=1}^{3L}\,{\eta }_{1}{\alpha }_{i}{\beta }_{i}$$

The term couples the different Majorana chains. Under the same basis as that of Eq. (14), it is written as ${ {\mathcal H} }_{NN}={\eta }_{1}\,{\rm{diag}}\{{\sigma }_{z},{\sigma }_{z},{\sigma }_{z}\}$, which describes the on-site energies of the electrons. The term breaks the chiral symmetry ${C}_{3}^{^{\prime} }$ in that ${C}_{3}^{^{\prime} }{ {\mathcal H} }_{NN}{C}_{3}^{^{\prime} -1}\ne -{ {\mathcal H} }_{NN}$. Then the total system belongs to the standard BDI class with one chiral symmetry *C*_3_. Its topological invariant corresponds to the number of Majorana zero modes on each boundary. As stated in the previous section, the topological invariant associated to the chiral symmetry *C*_3_ counts the difference of the numbers of *α*- and *β*-type Majorana zero states and it may not equal the number of zero-energy states. The above term gaps every pair of *α*- and *β*-type Majorana zero modes, thus now the topological invariant always equal the number of zero-energy states.

Next we consider a NNN hopping terms of Majorana fermions, which is described by,

$${H}_{NNN}=i\sum _{i=1}^{3L}\,{\eta }_{2}({\alpha }_{i}{\alpha }_{i+1}+{\beta }_{i}{\beta }_{i+1}\mathrm{).}$$

Under the same above basis, it is written as,

$${ {\mathcal H} }_{NNN}=2i{\eta }_{2}(\begin{array}{ccc}0 & I & -I{e}^{-ik}\\ -I & 0 & I\\ I{e}^{ik} & -I & 0\end{array})\mathrm{.}$$

The term is imaginary and breaks the time-reversal symmetry. The total system only has particle-hole symmetry underlying the superconductor, which is $P={C}_{3}{\mathscr{K}}$. So the system belongs to *D* class of the 1D topological classification. Its topological invariant is a *Z*_2_ integer, which means that we can only have 1 or 0 zero-energy state. The reduction of the topological invariant from *Z* to *Z*_2_ is due to the NNN hopping terms. It gaps every two Majorana zero modes, thus the cases with even (odd) number of Majorana zero modes become identical and the topological invariant changes to *Z*_2_ integer.

**Figure 5:**
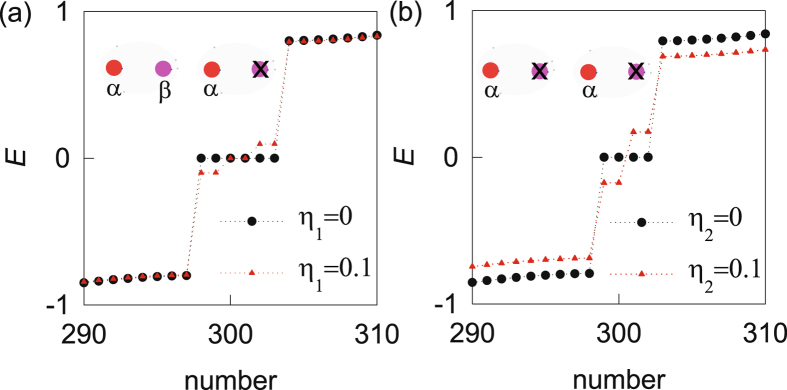
The open energy spectrum for the case of three Majorana chains. (**a**) The effect of NN hopping term in the topological phase with two *α* and one *β* Majorana zero modes at each end. (**b**) The effect of NNN hopping term in the topological phase with two *α* Majorana fermions at each end. The parameters in (**a**) are: ${t}_{1}^{^{\prime} }=0.3,\,{t}_{2}^{^{\prime} }=0.9$ and in (**b**) are: ${t}_{1}^{^{\prime} }=0.9,\,{t}_{2}^{^{\prime} }=0.3$, *η*_1_ = 0.1. The other parameters are *t*_1_ = 0.2, *t*_2_ = 1, ${t}_{1}^{^{\prime\prime} }=0.4,\,{t}_{2}^{^{\prime\prime} }=0.8$. The insets schematically show the left boundary zero modes in the absence of the symmetry breaking terms.

To be specific, we plot the open energy spectrums with the symmetry breaking terms. In Fig. 5(a) we start from a topological phase with two *α* and one *β* Majorana zero modes at each end, when there are six zero-energy modes. After adding the NN hopping term, one *α* and one *β* Majorana zero modes at each end are gapped and only one pair of Majorana zero modes remain. In Fig. 5(b) we start from a topological phase with two *α* Majorana zero modes at each end. After adding the NNN hopping term, they are gapped. The results are consistent with our previous analysis. The NN and NNN hopping terms gap every two Majorana zero modes and at most one Majorana zero mode persists on each end. So the topological invariant is reduced from *Z* to *Z*_2_, which corresponds to the change of the topological class from BDI to D. Thus we present an intuitive explanation of the *Z*_2_ nature of the topological invariant in the D class.

## Conclusions

We present a systematic method to construct 1D topological superconductors in BDI class by simply replacing fermion operators with Majorana ones in 1D topological insulators. Arbitrary winding numbers are realized by interpolate multiple such Majorana chains. Interestingly the combined systems belongs to BDI class, but have two kinds of chiral symmetries. One of the associated winding numbers counts the number of zero-energy modes, while the other counts the difference of the numbers of different type Majorana zero states. The physics meaning of the latter one is the destruction of the spatially intertwined Majorana zero states, which induces topological superconductors with coexistence of fractional charged and Majorana zero states in the systems with odd number of chains. Finally by introducing the symmetry breaking terms, an intuitive explanation of the reduction of the topological invariant is presented.

The constructed topological superconductors with arbitrary winding numbers can be used to study the effects of disorder or interactions on the topological classification^37^. They can describe 1D interacting Majorana models under the mean-field approximation^38^. Experimentally the periodically modulated Majorana chains can be engineered using Abrikosov lattice of vortices in the surface of a strong topological insulator coated with ordinary superconductors^24, 25, 39^.
